# Viral Metagenomics Reveals Widely Diverse Viral Community of Freshwater Amazonian Lake

**DOI:** 10.3389/fpubh.2022.869886

**Published:** 2022-04-25

**Authors:** Wylerson Guimarães Nogueira, Bruna Verônica Azevedo Gois, Kenny da Costa Pinheiro, Andressa de Oliveira Aragão, Ana Lídia Cavalcante Queiroz, Artur Luiz da Silva, Adriana Carneiro Folador, Rommel Thiago Jucá Ramos

**Affiliations:** ^1^Department of Biochemistry and Immunology, Federal University of Minas Gerais, Belo Horizonte, Brazil; ^2^Institute of Biological Sciences, Federal University of Pará, Belém, Brazil; ^3^Laboratory of Genomic and Bioinformatics, Center of Genomics and System Biology, Federal University of Pará, Belém, Brazil

**Keywords:** Amazon, Lake Bolonha, freshwater, viruses, bacteriophages, cyanophages, virome, metagenomics

## Abstract

Despite the importance of understanding the ecology of freshwater viruses, there are not many studies on the subject compared to marine viruses. The microbiological interactions in these environments are still poorly known, especially between bacteriophages and their host bacteria and between cyanophages and cyanobacteria. Lake Bologna, Belém, capital of the Brazilian State of Pará, is a water source that supplies the city and its metropolitan region. However, it remains unexplored regarding the contents of its virome and viral diversity composition. Therefore, this work aims to explore the taxonomic diversity of DNA viruses in this lake, especially bacteriophages and cyanophages, since they can act as transducers of resistance genes and reporters of water quality for human consumption. We used metagenomic sequencing data generated by previous studies. We analyzed it at the taxonomic level using the tools Kraken2, Bracken, and Pavian; later, the data was assembled using Genome Detective, which performs the assembly of viruses. The results observed here suggest the existence of a widely diverse viral community and established microbial phage-regulated dynamics in Lake Bolonha. This work is the first ever to describe the virome of Lake Bolonha using a metagenomic approach based on high-throughput sequencing, as it contributes to the understanding of water-related public health concerns regarding the spreading of antibiotic resistance genes and population control of native bacteria and cyanobacteria.

## Introduction

Amazonia is a 10 million years old unrivaled nest of biodiversity that reigns over South America; from bird-eating spiders to emperor tamarins to pink river dolphins, biologists find a new species every other day (1). Its ecosystems are essential for biodiversity preservation, climate regulation, energy production, and food and water security. The Amazon has a vital role in controlling zoonotic diseases and vector-borne and water-borne infections (2, 3).

Despite its importance, policies, laws, agreements, funds, and practical actions focused on Amazon protection have been weakened in Brazil, encouraging deforestation and culminating in losing about 20% of the original Amazon forest cover in Brazil by 2019 (4). The association between anthropogenic action in the Amazon rainforest, eutrophication of its water bodies, climate change, and alterations in vector dynamics, human migration, genetic changes in pathogens, and the poor social and environmental conditions in many Latin-American countries serve as an opportunity for the emergence and re-emergence of human infectious diseases in Brazil and other Amazonian countries (5).

Amazonian fauna hosts a vast diversity of well-known pathogens and many other potential new or even unknown pathogens (6–11). This abundance of microorganisms indicates that the emergence of new infections from the forest is a constant threat to human health, particularly favorable to water-borne diseases due to anthropogenic activities nearby freshwater sources, such as ponds, rivers, basins, and lakes (12–14).

Lakes close to urban areas are increasingly changing their ecosystem as human population expansion occurs and commercial, recreational, and residential uses increase (15). The eutrophication process offers particular conditions for the replication of viruses, as environments of this type seem to provide high viral activation and hypothetically control host abundance, respiration, and production (16).

Regarding lake environments, little is known about the ecology of the freshwater virus when compared to marine viruses (17). Most are bacteriophages or human and other animal viruses, but plant viruses are also identified (18). Bacteriophages can play an essential function in the aquatic ecosystem as they can contribute to the acquisition and spread of antibiotic resistance genes (ARGs) (19). Some studies have shown ARGs-carrying phages to be abundant in many environments, especially those impacted by anthropogenic activities (20–24), which demonstrates that these types of viruses are relevant to local microbial ecology (20).

Like bacteriophages, another essential group of DNA viruses is cyanophages, which infects cyanobacteria and have a similar morphology (25). Being abundant in both fresh and saltwater, they play an essential role in modulating cyanobacterial populations and preserving water quality (25, 26). Also, cyanophages are abundant in aquatic environments and play a fundamental role in flowering dynamics, including growth regulation and photosynthesis of cyanobacteria (27). However, unlike bacteriophages, it has many genera of possible hosts; therefore, freshwater cyanophages can be classified according to the taxonomy of their host organisms (28).

Given the need to study the diversity of different environments, techniques have been developed, such as viral metagenomics, also known as virome. This technique allows the study of various viruses from environmental samples (29). In this way, metagenomics and next-generation sequencing (NGS) have demonstrated considerable genetic complexity and inter-species and intra-species interaction by exploring viral populations both in aquatic environments and within the human microbiome (30, 31). However, despite increasing studies using the technique, there are still significant gaps in the virome databases. It has been estimated that 1,031 viral particles are infecting bacterial populations. However, <2,200 double-chain DNA virus (dsDNA) and retrovirus genomes are deposited at the National Center for Biotechnology Information (NCBI), compared to more than 45,000 bacterial genomes (32).

In order to understand the relationships that may exist between human actions and the emergence of new diseases from water sources in the Amazon, it is crucial to comprehend the role and dynamics displayed by the present viruses inside the local community. Therefore, our objective was to identify and describe the diversity of DNA viruses through viral metagenomics analyses in Lake Bolonha, especially those that have bacteriophagic and cyanophagic behavior, thus contributing to the future handling of water-borne diseases associated with resistance to antimicrobials of public health concern.

## Materials and Methods

### Sample Collection

**Figure 1:**
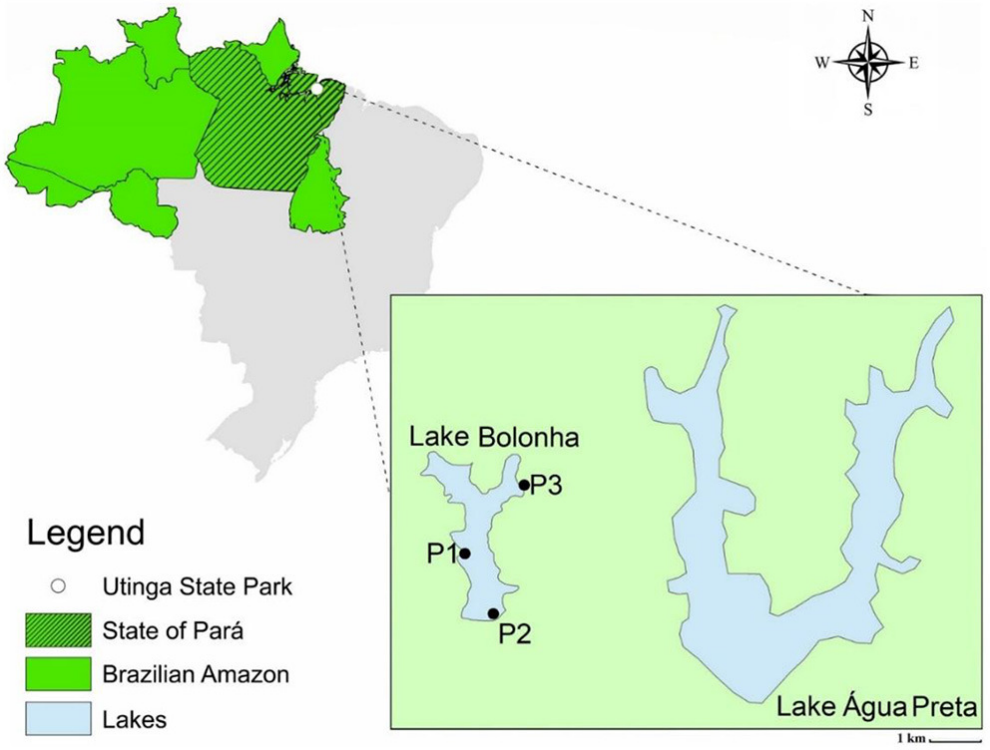
Map of the lake area and identification of sampling points (P1, P2, and P3). Alves et al. (33).

All sequencing data employed in this study were generated previously by Alves et al. (33). The water samples were collected in January of 2017 at Lake Bolonha, Belém-PA, at three different points, namely: P1 (S 01°25.530″ W 048°26.043″), upstream of the Water Treatment Plant uptake; P2 (S 01°25.530″ W 048°26.018″) in the morning-glory spillway that supplies other water treatment substations, and P3 (S 01°24.992″ W 048°25.785″) in the channel connecting the lakes Água Preta and Bolonha (Figure 1). The assessment of water quality, DNA extraction, and Total Community DNA (TC-DNA) metagenomics sequencing by the Ion Proton^TM^ platform are described in Alves et al. (33).

### Metagenomic Dataset

The raw metagenomic data used in this work is currently available at SRA/NCBI database under the access numbers SRR8893560 (P1), SRR8893561 (P2), and SRR8893559 (P3) (Table 1). This data can also be found at www.ncbi.nlm.nih.gov/sra/PRJNA506429. It is important to note that the data employed here was generated through high-throughput metagenomic sequencing of environmental samples using the Total Community DNA approach. However, we only included in our analyses the viral portion of the metagenomic dataset for this work being the virome our aimed subject of study.

**Table 1:** General information about the metagenomic dataset used in this work.

**SRA accession number**	**Sample code**	**Number of spots**	**Number of bases**	**Size**	**Study**	**Sequencing technology**
SRR8893560	P1	16,671,734	2.3 Gb	1.7 Gb	Freshwater Metagenome	Ion Proton™
SRR8893561	P2	16,278,151	2.2 Gb	1.7 Gb	Freshwater Metagenome	Ion Proton™
SRR8893559	P3	12,236,522	1.7 Gb	1.3 Gb	Freshwater Metagenome	Ion Proton™

### Taxonomy Analysis

The raw data was used to perform the taxonomic analysis through the Kraken2 tool (34) with the parameter “–download-library viral” to download the complete viral sequences of RefSeq and classify the reads regards its taxonomy.

### Assessment of Viral Diversity

The output generated by Kraken2 was submitted to the tool Bracken (35), using abundance and diversity to generate more accurate estimations on the viruses genus and species levels. The input parameters were “${CLASSIFICATION_LVL} = ‘S' (Species)” and “input data = kraken2 output (report).” All other parameters were set as default. Later, the results were displayed with the Pavian tool (36), which allows comparing the taxonomic classifications obtained by Kraken2 and Bracken and presenting abundance estimations in several samples. Veen diagrams were generated using the web-based tool InteractiVenn (37).

### Viral Metagenome Assembly

The online tool Genome Detective (38) was used in default parameters to assemble the sequencing data and classify the contigs formed into their respective taxa, identified using metaSPAdes software for single-end reads (39).

## Results

### Virome Assembly

The viral portion of the raw metagenomic data was assembled to prepare the data for the classification of their respective taxon per each of the freshwater samples (Table 2). The species with the highest percentage of coverage after assembly were Cyanophage KBS-S-2A (P1) with 25.37% and 69.66% identity, *Cladosporium fulvum* T-1 virus (P2) with 23.14% coverage and 56.90% identity, and Cyanophage KBS-S-2A (P3) with 20.65% coverage and 71.18% identity.

**Table 2:** List of samples (P1, P2, and P3) and their respective number of reads obtained after the sequencing, after quality control, the number of reads associated with viruses, and the number of reads used for coverage depth.

**Sample code**	**Raw data**	**After control quality**	**Reads associated with viruses**	**Average depth of assembly**
P1	16,671,734 reads	8,410,372 reads	202,318 reads	5,359 reads
P2	16,278,151 reads	8,339,898 reads	44,877 reads	4,067 reads
P3	12,236,522 reads	6,299,043 reads	131,370 reads	2,217 reads

### Viral Diversity of Lake Bolonha

**Figure 2:**
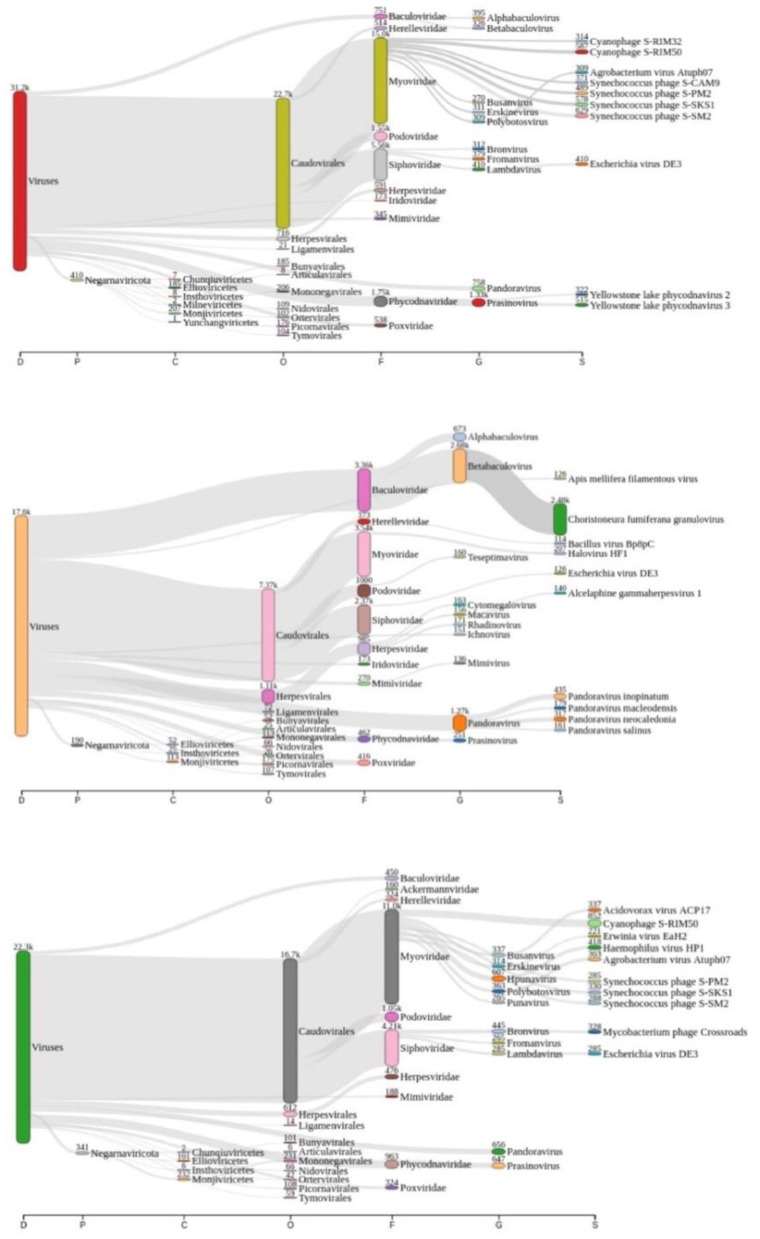
Sankey diagram display of the taxonomic diversity found at samples P1 (red “viruses” tag), P2 (coral “viruses” tag), and P3 (green “viruses” tag), respectively.

Taxonomic analysis performed by Kraken2 revealed that there might be more than 3.500 distinct species of viruses in Lake Bolonha. A Sankey diagram is represented summarizing taxonomic diversity at samples P1, P2, and P3, respectively, in Figure 2. The complete data from the taxonomic analysis comparison between each sample site is reported as Supplementary Tables S1–S3.

**Figure 3:**
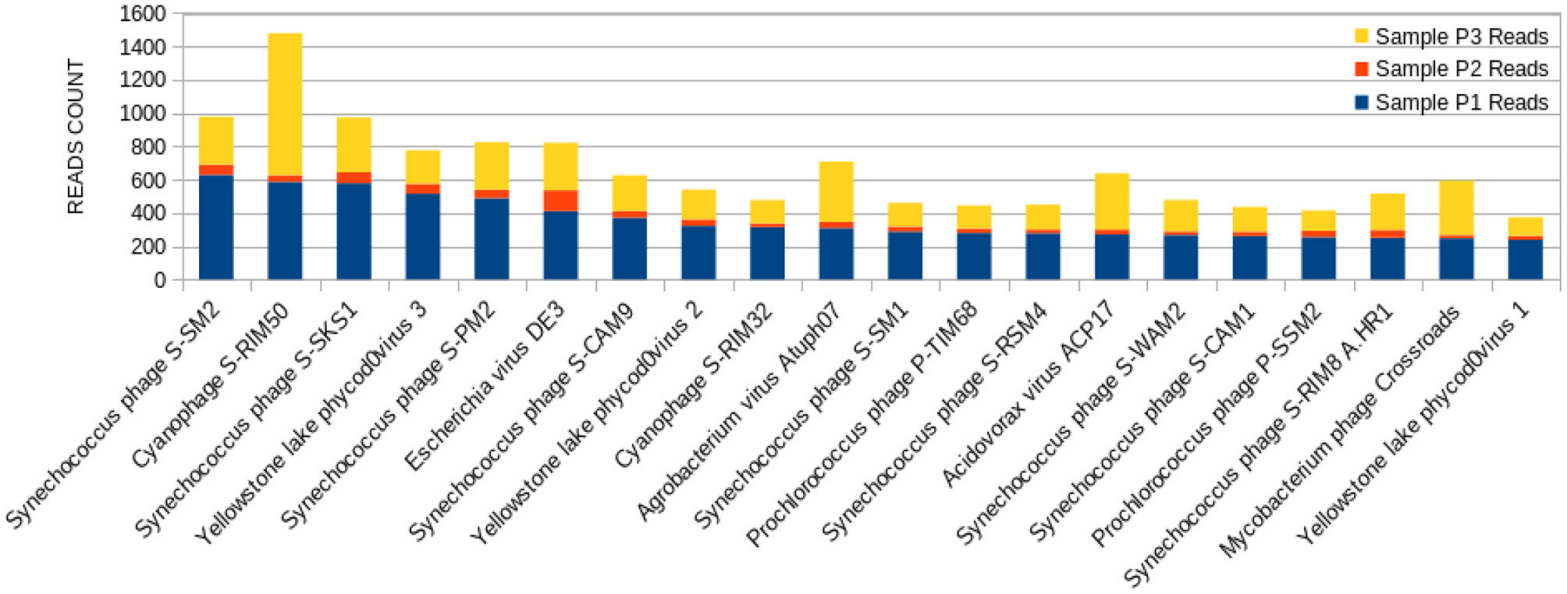
Bar graph showing the distribution of reads for each top 20 virophages found in sample P1, plotted against their respective representations on samples P2 and P3.

**Figure 4:**
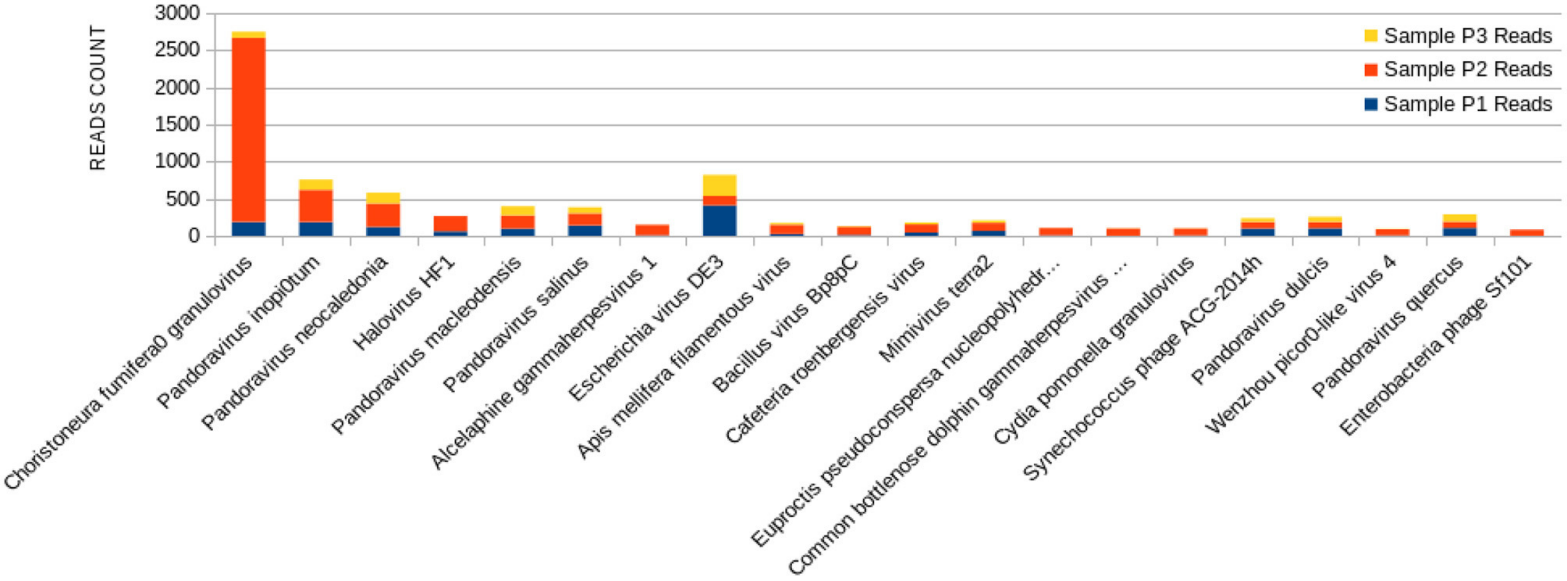
Bar graph showing the distribution of reads for each top 20 virophages found in sample P2, plotted against their respective representations on samples P1 and P3.

**Figure 5:**
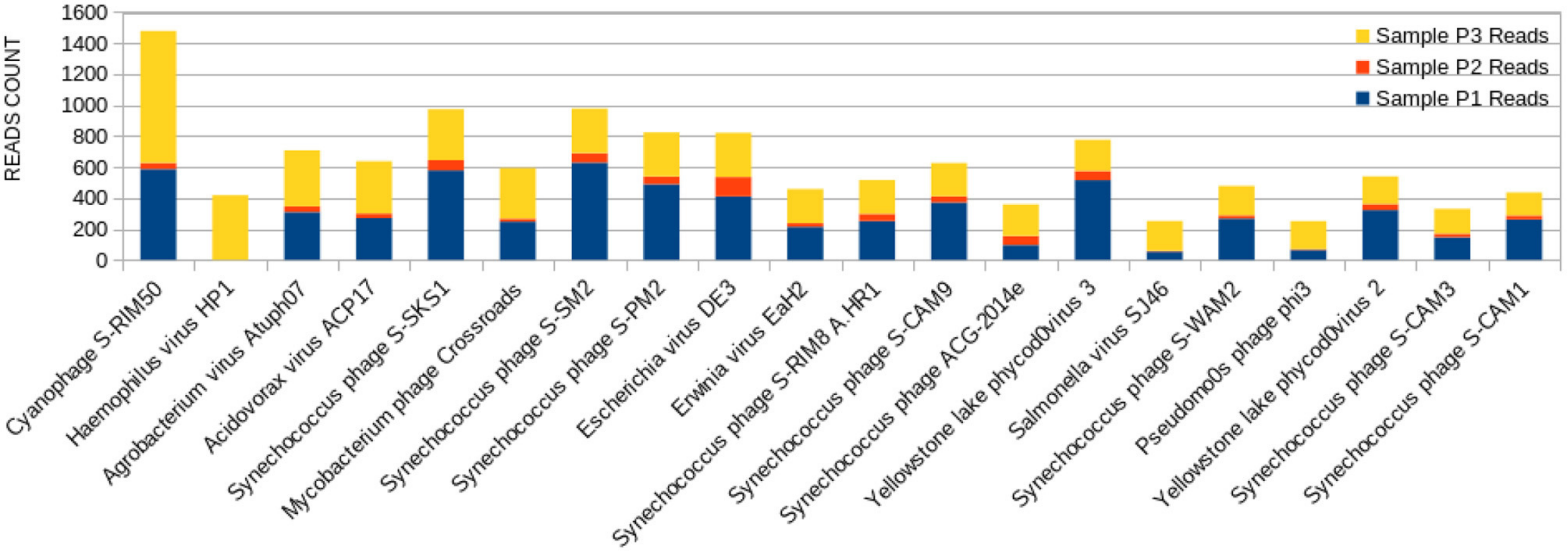
Bar graph showing the distribution of reads for each top 20 virophages found in sample P3, plotted against their respective representations on samples P1 and P2.

**Figure 6:**
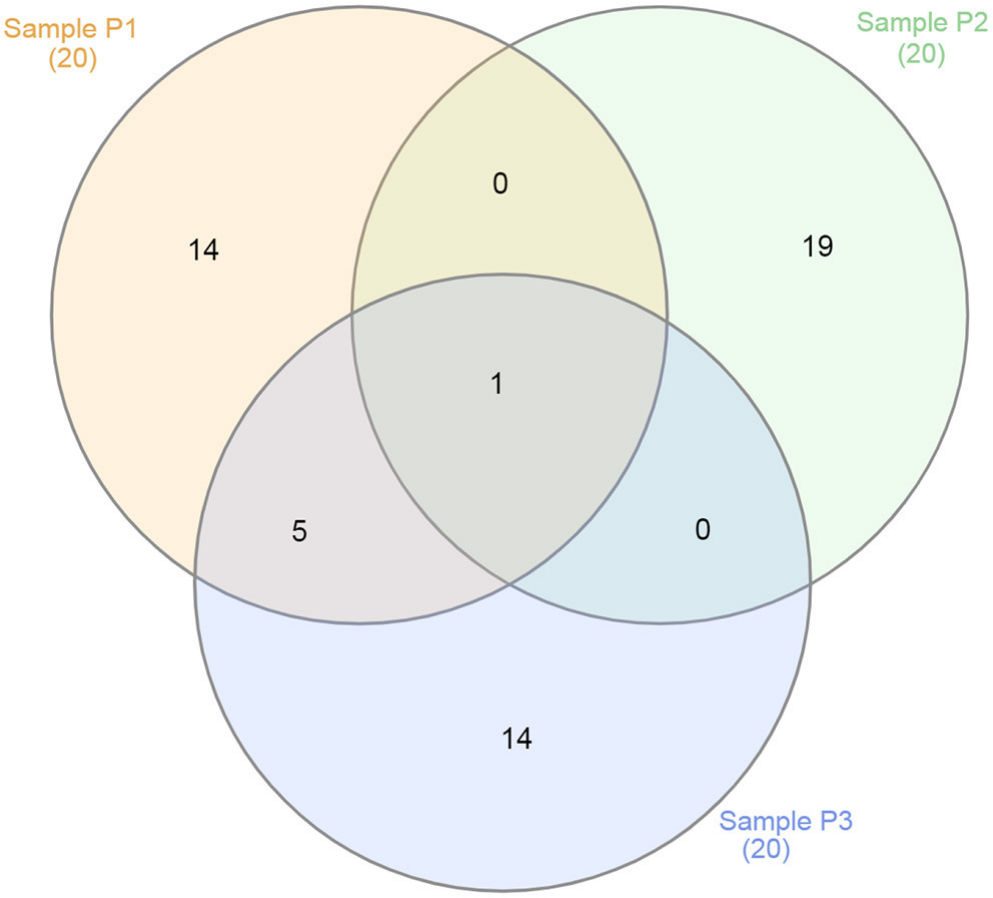
Venn diagram showing the overlap between the top 20 viruses for samples P1 (in red), P2 (in green), and P3 (in blue).

The 20 species with a higher reads count in all freshwater samples are represented in Figures 3–**5**. The number of reads found for each virus on this top selection was compared with their respective number of reads on the other two samples from this study. Also, a Venn diagram showcasing the overlap between these top 20 most represented viruses for samples P1, P2, and P3, is displayed in **Figure 6**. A similar diagram for the complete dataset of the identified viruses is reported as Supplementary Figure S1.

The sample P1 presented an overall abundance of *Synechococcus* phage, a cyanophage (Figure 3). We obtained the highest reads for a single virus in sample P2 for *Choristoneura fumiferana granulovirus* (Figure 4). The sample P2 also presented a much more significant amount of the genus Pandoravirus and Mimivirus compared to other collection sites. *Haemophilus* phage HP1 was only observed on sample P3 (Figure 5).

## Discussion

Aquatic ecological environments have a broad range of viruses that are crucial in controlling bacterial communities and regulating biogeochemical cycles (40, 41). While most of the literature on water virome has concentrated on viruses in marine waters (42, 43), previous research indicated that freshwater harbored specific viral communities distinct from other aquatic environments (44). Other studies have described the characterization of freshwater viromes in ballast water (45), sewage (46, 47), lakes (48, 49), river estuaries, where marine and freshwater mix (50–52), and long rivers (53). In the present study, we identified the diversity of the viral community for three different metagenomic samples from the Lake Bolonha, obtained by the previous work of Alves et al. (33), to acquire a vivid understanding of this Amazonian lake water system.

According to Alves et al. (33), the Amazonian vegetation on its shore characterizes Lake Bolonha and the propagation of large plants under its surface, resulting in eutrophication. Also, it presents increased phosphorus and total nitrogen values in physical-chemical analysis and a high fecal coliform rate (33). In this study, we noted the significant presence of cyanophages, mainly at P1 and P3 (Figure 2). This finding contributes as an essential indicator of the interference of these phages in the environment, known for their ability to perform photosynthesis by consuming oxygen and their potential for binding to nitrogen and producing toxins (54). Nitrogen or phosphorus supplies and reduced growth rate and biomass may naturally limit freshwater ecosystems, including those involving cyanobacteria accumulation (55).

When it comes to viral abundance, taxonomic analysis reveals that a plethora of more than 3.500 distinct viruses is present at freshwater Brazilian Lake Bolonha, an area of environmental preservation. It is possible to observe that the phages with a higher abundance of reads in P1 and P3 have a lower distribution among their P2 (Figures 3–5), which can be explained by the proximity between sampling sites P1 and P3. The alpha diversity observed in the raw sequencing data of Table 1 of Alves et al. (33) also shows significant sample abundance in P1 and P3, which could also be related to this observation.

The diversity and abundance imbalance of essential viruses, such as bacteriophages and cyanophages, can cause significant changes in the aquatic ecosystem. Some of these phages can mediate the transduction of resistance genes between bacteria, which can provide evolutionary advantages to microorganisms and affect the water quality (33). In addition, understanding this viral community's diversity could help prevent the spread of antimicrobial resistance elements and circumvent possible future multi-resistant pathogen epidemics.

Although all samples came from the same lake and environment, the overlap of viruses best represented at each sampling site showed a vast distinction when considering its top 20 viruses (Figure 6). For instance, only Escherichia phage DE3 was at the top 20 viruses for all three samples. At the top, only shared by samples P1 and P3 were five viruses: *Mycobacterium* phage Crossroads; Yellowstone lake phycodnavirus 3; *Agrobacterium* phage Atuph07; Yellowstone lake phycodnavirus 2; and Acidovorax virus ACP17. Moreover, sample P2 had no virus shared exclusively with sample P1 or P3 at its top 20. That observation shows the diversity of viral entities present in a single environment, such as Lake Bolonha. It highlights choosing different and representative sites when studying an environmental microbiome.

The sample P1 presented an abundance of *Synechococcus* phage (Figure 3). This phage frequently infects cyanobacteria of the *Synechococcus* genus in diurnal patterns of infection due to the photosynthetic activities of its host (56). This infection affects their population dynamics by killing part of this cyanobacteria population daily, estimated between 0.005 and 30% per day (27, 57, 58). In addition, it has been described that such cyanophages play a vital role in the diversity and evolution of their host cyanobacteria (18, 32, 54, 59–62).

Amongst the abundant species, the cyanophage S-RIM50 was found in P1, which could infect cyanobacteria of the genus *Synechococcus* (63). The abundance of cyanophage S-RIM50 has been reported in both fresh and seawater (26). Cyanophages such as the cyanophage S-RIM50 and *Synechococcus* phage are abundant in freshwater environments. They have been isolated from various freshwater reserves, including lakes, ponds, streams, and sewage points (27, 56, 58). They have an essential contribution to maintaining the cyanobacterial community and the preservation of water quality (25).

*Synechococcus* phages, present in both samples P1 (Figure 3) and P3 (Figure 5), can be associated with health problems such as multiple sclerosis. This phage expresses proteins containing consensus peptide stretches that are highly homologous to the products of 16 autoantigens related to multiple sclerosis susceptibility genes (64). Other viruses associated with multiple sclerosis, such as the Epstein-Barr virus, have also shown this behavior, and the bacteriophage *Synechococcus* has been identified as a new relevant contributor to this phenomenon. Its cyanobacterial host prefers a temperate climate, indicating that the ecology of this cyanophage is consistent with the overall distribution and epidemiology of multiple sclerosis (63).

Yellowstone Lake phycodnaviruses, a double-stranded DNA virus that infects algae, and *Escherichia* phage DE3 on the sample P1 were also observed (Figure 3). A more significant number of reads associated with *Shigella* phage SfIV was observed compared to samples P2 and P3 (Figure 3). It is important to mention a relationship with the possible environmental presence of the *Shigella* bacterial host, responsible for causing intestinal infection followed or not by fever, colic, and diarrhea with blood and mucus (65). This observation demonstrates the importance of this study in characterizing the environmental conditions as a possible source of information for public health.

The P2 collection point presented many *Choristoneura fumiferana granulovirus* (Figure 4), part of the *Baculoviridae* family (66). *Halovirus* HF1, responsible for infecting members of the *Halobacteriaceae* family (67), was also identified (Figure 4). Interestingly, a small number of reads (25 reads) associated with the species *Diplodia scrobiculate* RNA virus 1 is only present in P2. As a preferable host, this phage has the endophytic fungus *Diplodia scrobiculate*, which primarily affects the genus *Pinus* spp. among other conifers (68), which would be odd to find on a water sample from a tropical locality.

A considerable amount of *Bacillus* phage Bp8pC has also been observed on sample P2 (Figure 4), which hosts the bacteria *Bacillus thuringiensis* and *Bacillus pumilus*. Both are of economic importance because they are used in agriculture as pest control bringing little harm to humans (69). The presence of the genus *Bacillus* in lake water may indicate the contamination of the water environment by different types of residues coming from the watershed to the lake (70).

Exclusively on P2, a much more significant amount of the genus Pandoravirus and Mimivirus was observed compared to the other collection points (Figure 4). Previous studies suggest a potential role of Mimivirus in respiratory pathology displayed during seroconversion in patients with pulmonary pneumonia. In addition, positive serology for Mimivirus is associated with increased duration of mechanical ventilation supported breathing and intensive care unit in patients with ventilator-associated pneumonia (71). Both genera are constituted of giant viruses and have Amoeba as their typical host (72–74). Pandoravirus has a size of about 1 micron and may resemble some types of bacteria. Their genome contains more than 100 distinct genes and can be twice as large as the Mimivirus genome, besides the fact that their genome is quite different compared to other known organisms (75).

*Haemophilus* virus HP1, a bacteriophage that infects the *Haemophilus influenzae* bacterium (76), was only observed at sample P3 (Figure 5). Its sampling site is located at the starting point of the channel connecting both Lakes Bolonha and Água Preta. It is crucial to note that what occurs at this site may in the future influence the environment of nearby Lake Água Preta.

Overall, these results denote the presence of a diverse viral community and suggest the existence of established regulation dynamics in the local microbial environment of Lake Bolonha, highly influenced by the bacteriophages and cyanophages that inhabit the location. The dispersion of those biological entities along the water distribution channels using Lake Bolonha as a water source and general eutrophic activity might contribute to the spread of minor genetic elements like ARGs and future unbalance the microenvironment of close by freshwater sources, such as Lake Água Preta.

## Conclusions

Given the importance of Lake Bolonha as a source of drinking water supply for the metropolitan region of Belém, the elucidation of the viral diversity from this environment is relevant to provide a better understanding of how its exploration can affect it. The results observed in this work indicate a widely diverse viral community, especially bacteriophages and cyanophages. These findings also suggest the existence of established micro-environmental dynamics in Lake Bolonha, possibly regulated by such phage entities. The dispersion of those viral beings bare similarity along the course of the lake, apparently more related the deeper they are into the lake (P1, P3) and the further away they are from the water evacuation sites to other treatment substations (P2). This study is the first-ever work to describe the virome of Lake Bolonha and, as such, contributes to the understanding of water-related public health concerns regarding the spreading of antibiotic resistance genes and population control of native bacteria and cyanobacteria.

## Data Availability Statement

The datasets presented in this study can be found in online repositories. The names of the repository/repositories and accession number(s) can be found below: https://www.ncbi.nlm.nih.gov/; https://www.ncbi.nlm.nih.gov/sra/PRJNA506429.

## Author Contributions

BG, KP, and RR designed the study. WN, BG, and KP compiled and curated the data, performed bioinformatic analysis, and interpreted the results. AF and RR supervised and administered the project and provided funding. WN and BG wrote the original draft and manuscript with input from KP, AA, AQ, AF, and RR. All authors critically reviewed the manuscript and approved the final version.

## Funding

We thank the Coordenação de Aperfeiçoamento de Pessoal de Nível Superior–CAPES and PROPESP/UFPA (Pró-Reitoria de Pesquisa e Pós-Graduação / Universidade Federal do Pará), the funding agencies FAPESPA (Fundação Amazônia de Amparo à Estudos e Pesquisas), and FAPEMIG (Fundação de Amparo à Pesquisa do Estado de Minas Gerais) for financial support on this work.

## Conflict of Interest

The authors declare that the research was conducted in the absence of any commercial or financial relationships that could be construed as a potential conflict of interest.

## Publisher's Note

All claims expressed in this article are solely those of the authors and do not necessarily represent those of their affiliated organizations, or those of the publisher, the editors and the reviewers. Any product that may be evaluated in this article, or claim that may be made by its manufacturer, is not guaranteed or endorsed by the publisher.

## Supplementary Material

Supplementary Figure 1 Venn diagram representation of overlapping viruses for Samples P1, P2, and P3 complete datasets.

Supplementary Table 1 Full report table of identified viruses from taxonomic analysis performed by Kraken2 for Lake Bolonha's metagenome Sample P1.

Supplementary Table 2 Full report table of identified viruses from taxonomic analysis performed by Kraken2 for Lake Bolonha's metagenome Sample P2.

Supplementary Table 3 Full report table of identified viruses from taxonomic analysis performed by Kraken2 for Lake Bolonha's metagenome Sample P3.

## Abbreviations

ARGs: Antibiotic resistance genes
NGS: Next-Generation Sequencing
dsDNA: double-chain DNA
NCBI: National Center for Biotechnology Information.
