# Supplementary figures and images for: Viral Metagenomics Reveals Widely Diverse Viral Community of Freshwater Amazonian Lake
Source: Front Public Health. 2022 Apr 25;10:869886. doi: 10.3389/fpubh.2022.869886 (PMC9081339; doi:10.3389/fpubh.2022.869886)

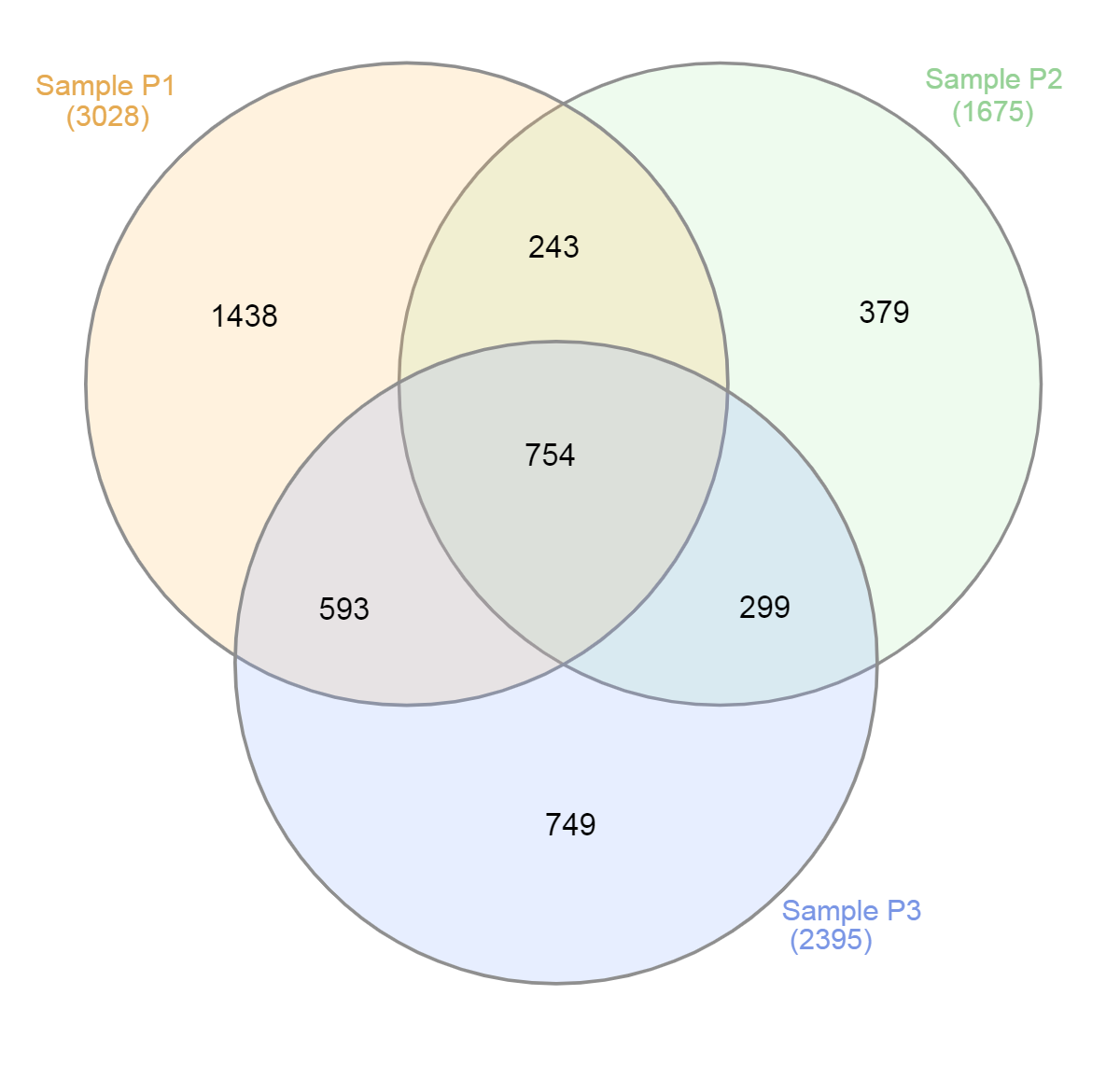

Supplement: Supplementary Figure 1 — Venn diagram representation of overlapping viruses for Samples P1, P2, and P3 complete datasets. [file Image_1.TIF]
